# Thermal Conductivity Enhancement of Cu‐ and Ag‐Decorated MWCNT Hybrid Nanofluids Synthesized via Probe Ultrasonication

**DOI:** 10.1002/open.70243

**Published:** 2026-06-08

**Authors:** S. Heshmatian, M. Aligholami, M. Akbari, S. Shafiei, I. G. Madiba, S. Azizi, M. Maaza

**Affiliations:** ^1^ UNESCO‐UNISA Africa Chair in Nanosciences‐Nanotechnology College of Graduate Studies University of South Africa Pretoria South Africa; ^2^ Department of Engineering Sciences and Physics Buein Zahra Technical University Buein Zahra Iran; ^3^ Nanosciences African Network Materials Research Dept. iThemba LABS/National Research Foundation of South Africa Somerset West South Africa

**Keywords:** hybrid nanofluids, multiwalled carbon nanotubes, probe ultrasonication, thermal conductivity, thermal management

## Abstract

Hybrid Ag/MWCNT and Cu/MWCNT nanofluids were synthesized in ethylene glycol using a surfactant‐free probe ultrasonication method and evaluated at ultralow solid concentration (0.001–0.01 wt%). TEM analysis indicated decoration of MWCNTs with Ag and Cu nanoparticles, while Raman spectroscopy indicated defect‐mediated interactions and structural perturbation of the functionalized MWCNT framework, as suggested by increased *I*_D_/*I*_G_ ratios. UV–Vis spectra showed Ag/MWCNT and Cu/MWCNT plasmonic bands at 380–420 and 300–360 nm, respectively, confirming dispersion in EG. At 0.001 wt%, periodic UV–Vis/zeta‐potential tests over 4 weeks indicated acceptable stability, with preserved spectra and negative *ζ* values ≥30 mV. Thermal conductivity measurements (20°C–70°C) revealed clear enhancement with temperature and metal concentration, reaching ≈5.5% for Ag/MWCNT–EG and ≈8% for Cu/MWCNT–EG at 0.01 wt% and 70°C. The superior performance of Cu/MWCNT–EG is likely associated with stronger interfacial interactions between Cu and carboxylated MWCNTs, enabling more efficient phonon–electron heat transfer pathways. Compared with similar carbon‐, metal‐, and hybrid‐based nanofluids commonly using ≥0.05–0.3 wt%, this work achieves measurable thermal improvement at 0.001–0.01 wt%, highlighting an effective, scalable, surfactant‐free platform for designing stable, low‐viscosity, high‐performance heat transfer fluids for high heat flux heat exchangers, electronic cooling applications.

## Graphical abstract

Surfactant‐free Ag/MWCNT–EG and Cu/MWCNT–EG hybrid nanofluids are prepared via probe ultrasonication at ultralow loadings. The nanofluids show stable dispersion at the lowest concentration and measurable thermal conductivity enhancement, reaching approximately 5.5% and 8% for Ag/MWCNT–EG and Cu/MWCNT–EG, respectively, highlighting their potential for low‐loading thermal management applications.

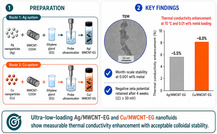

## 1 Introduction

Efficient heat removal remains one of the main design challenges in many modern technologies. From compact power electronics and electric‐vehicle batteries to concentrated solar power [1] and industrial heat exchangers, thermal management is limited by the low thermal conductivity of conventional heat‐transfer fluids (HTFs) such as water, ethylene glycol (EG), and various oils. These base fluids typically have a conductivity lower than 0.6 W·m^−1^·K^−1^ [2]. As a result, designers must oversize cooling systems, increase pumping power, and tolerate larger temperature differences. Such solutions increase cost, weight, and energy consumption, while reducing overall efficiency and reliability.

The introduction of nanofluids—that is, containing a small amount of well‐dispersed nanoparticles, provided a promising pathway for improving heat transport beyond the limits of pure liquids. Choi and Eastman [2] first proposed that adding highly conductive nanoparticles could enhance the overall thermal conductivity of a fluid in ways not predicted by classical mixture models. Their pioneering work opened an entirely new field that has since expanded to include numerous nanoparticle types (metals, metal oxides, carbides, nitrides, and carbon‐based materials), particle shapes (spheres, rods, tubes, and flakes), and even hybrid combinations of different materials [2, 3, 4, 5, 6, 7, 8, 9, 10, 11, 12].

Nanofluids are engineered colloidal suspensions of nanoparticles (1–100 nm) in conventional fluids such as water, EG, or oil. Their enhanced thermal performance has been attributed to several possible, and still debated, physical mechanisms: (i) the very high intrinsic thermal conductivity of the dispersed phase, (ii) improved interfacial heat transfer due to surface functionalization, (iii) possible microconvection associated with Brownian motion of nanoparticles, (iv) the formation of continuous percolation networks, and (v) liquid layering at the solid–liquid interface that modifies phonon transport. However, the relative contribution of each mechanism remains debated and depends strongly on nanoparticle type, concentration, base fluid, surface chemistry, and temperature. Although each mechanism's contribution depends on particle type and processing, all studies agree that stability, resistance to agglomeration, and sedimentation, is essential for reproducible and reliable measurements [13, 14]. Unstable nanofluids can show artificially high conductivity immediately after preparation but rapidly lose performance due to particle settling. Hence, achieving a stable suspension is a central goal in nanofluid research.

Over the past two decades, a large number of studies have sought to optimize synthesis and stabilization methods. Techniques range from one‐step synthesis (e.g., laser ablation in liquids, radiolysis, or chemical reduction directly in the base fluid) [15, 16, 17, 18, 19, 20, 21, 22, 23, 24] to two‐step dispersion of presynthesized particles using surfactants, polymers, or pH control [25, 26, 27, 28, 29, 30, 31]. One‐step approaches produce clean particle surfaces but are often expensive or difficult to scale. Two‐step routes are easier and flexible but may suffer from particle agglomeration if surfactants are not well chosen. Probe ultrasonication has recently emerged as an effective and scalable method that uses acoustic cavitation and high shear to break clusters and uniformly disperse nanoparticles without surfactants. In systems containing presynthesized nanoparticles, probe ultrasonication can promote dispersion and interfacial attachment of nanoparticles onto carbon structures through localized high‐energy microjets and shear forces [30, 31, 32, 33, 34, 35, 36].

**FIGURE 1:**
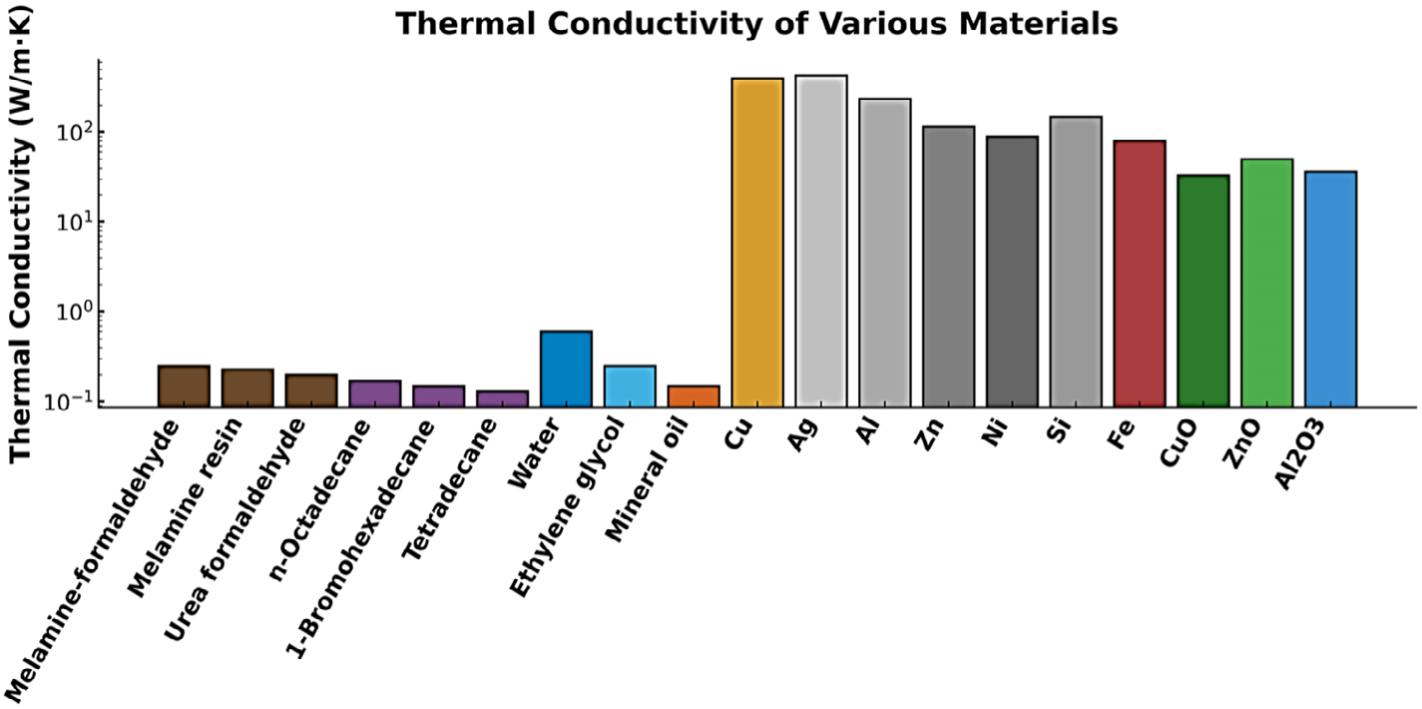
Comparative thermal conductivity of some organic materials, metals, metal oxides, and heat‐transfer fluids.

Figure 1 compares the thermal conductivity of typical organic liquids, metals, and metal oxides, showing the significant contrast that motivates the use of highly conductive nanoparticles in conventional HTFs. For example, silver has a conductivity of about 429 W·m^−1^·K^−1^ and copper about 398 W·m^−1^·K^−1^, compared with <0.6 W·m^−1^·K^−1^ for water or EG. This large conductivity contrast supports the motivation for hybrid nanofluids, where highly conductive metals provide efficient electronic heat‐transfer pathways, while carbon nanostructures such as MWCNTs can act as thermally conductive networks that improve dispersion and interfacial heat transport (see Figure 1).

Early nanofluids mostly used a single type of nanoparticle. Later, hybrid nanofluids (which combine two or more types of nanoparticles) were introduced to achieve synergistic effects. For instance, combining a metal with a carbon material can merge electronic and phononic heat‐transfer paths while maintaining good dispersion [18, 30, 37, 38, 39, 40, 41, 42].

Carbon nanotubes (CNTs) and graphene are widely recognized for their extremely high intrinsic thermal conductivity (>2000 W·m^−1^·K^−1^) and large aspect ratio. Even at concentration below 0.05 wt%, they can form long‐range thermally conductive networks. Metals such as Cu and Ag provide electron‐dominated heat transport and raise the effective thermal conductivity even at low concentrations. Decorating CNTs with metals or metal oxides reduces the spacing between high‐thermal conductivity domains and creates strong interfacial bonds, thus enhancing both conductivity and stability [4, 5, 17, 18].

Hybridization also helps overcome a common problem in pure metallic nanofluids: poor colloidal stability. While metallic particles conduct heat well, they tend to oxidize or cluster under thermal cycling. Anchoring them onto functionalized MWCNTs (for instance, COOH‐modified MWCNTs) improves dispersion and prevents van der Waals‐driven aggregation.

Previous studies have investigated a wide range of metal‐, oxide‐, and hybrid‐based nanofluids, typically reporting thermal conductivity enhancements of 5%–20% depending on nanoparticle type, base fluid, and operating conditions [6, 7, 43, 44, 45, 46, 47, 48, 49, 50, 51, 52, 53]. However, these improvements are generally achieved at relatively high nanoparticle concentrations (≥0.05 wt%) and often require surfactants or chemical stabilization, which may increase viscosity and compromise long‐term stability. Therefore, achieving meaningful thermal conductivity enhancement at ultralow concentrations while maintaining colloidal stability remains a key challenge in nanofluid research.

Table 1 summarizes the thermal conductivity enhancement and stability characteristics of representative nanofluids reported in the literature, including metal‐, oxide‐, and hybrid‐based systems. A detailed comparison with the present work is provided in the Results section to highlight its relative performance.

**TABLE 1:** Benchmark studies of low‐to‐moderate‐concentration Cu and Ag and related metal–carbon hybrids, their thermal conductivity enhancement, and stability.

Ref.	NPs	**Base fluid**	**Size, nm**	**Concentration, %**	***T*, °C**	***k*** **enhancement, %**	**Stability** **/notes**
[48]	Ag, AlN, MWCNT	Water	Ag: 20–40; AlN: 50–80; MWCNT: 10–20	0.01–0.03 wt%	20–45	≈5–9	Surfactant‐dependent; SDBS/CTAB best for Ag/AlN; SDS/GA best for MWCNT
[49]	Ag/MWCNT	Water	≈25	≤0.1 wt%	20–50	0.16–8.02 (vs. undecorated MWCNT NF)	Slight aggregation after 72 h
[50]	Ag/MWCNT	Water	—	MWCNT 0.05 wt% + Ag 3 wt%	≈25	14.5	Stable ≈2 weeks; low viscosity variation
[51]	Ag (polyol)	EG	—	≤few wt% (low φ)	—	≈3	stable > 2 weeks Polymer‐stabilized
[53]	CuO/Gr	Water	—	0.01–0.05 wt%	30–80	4% at 30°C; 7.16% at 80°C	Rhamnolipid biosurfactant improves stability
[54]	Fe_3_O_4_/MWCNT	Water/EG	Fe_3_O_4_: 20–30; Fe_3_O_4_@MWCNT: 46–68	0.05–0.2 wt%	25	2.17; 5.14; 5.94	Zeta potential −36 to −58 mV; highly stable
[55]	Gr/SiO_2_	Water‐based cutting fluid	N/A	Not stated	Not stated	18.1	Polymer‐stabilized; improved thermal conductivity
This work	Cu/MWCNT Ag/MWCNT	EG	Cu: 30 Ag: 20 MWCNT ≈ 30–50	0.001–0.01 wt% (MWCNT 0.001 wt%)	20–70	~5.5–8 at 70°C	Stable ≥ 1 month

The studies were selected because they report low‐to‐moderate‐concentration metal‐, metal oxide‐, carbon‐, or hybrid nanofluids with available thermal conductivity enhancement and/or stability data relevant to comparison with the present Ag/MWCNT–EG and Cu/MWCNT–EG systems. For consistency, all concentration values in Table 1 are presented in wt% to allow clearer comparison with the present ultralow‐concentration formulations.

Despite considerable progress, three key gaps directly motivate the present study:

1. Direct comparative studies: Few studies have synthesized Cu/MWCNT and Ag/MWCNT nanofluids under identical conditions, making it difficult to evaluate metal‐specific interfacial effects.
2. Limited focus on ultralow concentration: Most studies employ nanoparticle concentrations above 0.1 wt%, which may increase viscosity, filter clogging, and sedimentation problems. Data on ultralow concentrations (0.001–0.01 wt%) remain scarce.
3. Limited long‐term stability assessment: Many reported nanofluids show stability only for a few days, while month‐scale observations under varying temperature conditions are rarely presented.

To address these limitations, a systematic comparison with existing nanofluid systems is incorporated in Table 1 and further discussed in the Results section. Accordingly, this work aims to prepare Cu/MWCNT–EG and Ag/MWCNT–EG nanofluids under identical surfactant‐free probe ultrasonication conditions, evaluate their thermal conductivity at ultralow loadings, and assess their dispersion stability using UV–Vis and zeta‐potential measurements.

Addressing these limitations requires a unified synthesis and testing framework, as used in the present study, to fairly assess how Cu and Ag decoration on MWCNTs influences both conductivity and stability. Probe ultrasonication simultaneously disperses MWCNTs and promotes the attachment of presynthesized Ag or Cu nanoparticles onto their surfaces. The cavitation energy breaks large clusters into smaller aggregates and promotes effective wetting between the nanoparticles and the base fluid. Our previous works on TiO_2_/MWCNT and Ag/MWCNT hybrids demonstrated that this approach yields long‐term stability at ultralow concentrations (0.001–0.01 wt%) [36, 37]. This motivates the current extension to Cu‐ and Ag‐decorated MWCNTs in an EG base fluid.

The heat‐transfer performance of nanofluids is also influenced by nanoparticle–fluid interfacial interactions, including interfacial resistance, Brownian motion, and thermophoresis, whose relative contributions depend on particle size, surface chemistry, base fluid, and temperature [3, 4, 5, 6, 7, 8].

Functionalization of MWCNTs with carboxyl (–COOH) groups improves their dispersibility in polar media like EG [36, 54]. When Cu or Ag nanoparticles are attached to these functional groups, the resulting metal–carbon interactions create additional pathways for heat flow. The probe ultrasonication process supplies intense localized energy that breaks weakly bound aggregates and promotes nanoparticle attachment onto the MWCNT surface. Maintaining the temperature below 30°C during sonication avoids overheating and preserves particle integrity.

Ultrasonication parameters such as power, frequency, pulse cycle, and duration determine the cavitation intensity and hence the final dispersion quality. Such details are essential for ensuring a reproducible microstructure and stability. Studies show that dispersion stability in nanofluids is strongly influenced by ultrasonication conditions, surface functionalization, surfactant use, and zeta potential, with |*ζ*| values greater than 30 mV commonly used as an indicator of electrostatic stabilization [35, 56, 57, 58, 59, 60, 61].

In this study, nanofluids are prepared in EG via a single surfactant‐free probe‐ultrasonication method. The key novelties are as follows:

1. **Extremely low concentration window:** Both hybrids were synthesized at only 0.001–0.01 wt% total solids (0.001 wt% MWCNTs), achieving measurable thermal conductivity enhancement (~5.5%–8% at 70°C). These concentrations are much lower than those used in most Cu or Ag nanofluid studies.
2. **High stability without surfactants:** The lowest‐concentration formulations showed acceptable month‐scale colloidal stability at room temperature, confirmed by UV–Vis absorption and zeta‐potential analysis. This stability exceeds most reported durations (typically 7–14 days [48, 49, 50, 51]).
3. **Comparable dual‐metal analysis:** Both Cu and Ag systems were processed under identical conditions, allowing a fair comparison of their dispersion quality and heat transfer behavior on the same MWCNT matrix.
4. **Scalable, clean synthesis:** The surfactant‐free ultrasonication route avoids organic residues that typically hinder conductivity and can be scaled up to liter‐level volumes.

These advances broaden the range of feasible formulations for stable, low‐concentration hybrid nanofluids tailored for energy‐efficient heat‐transfer applications.

This article describes the synthesis, morphological characterization (transmission electron microscopy [TEM]), structural analysis (Raman spectroscopy), and dispersion assessment (UV–Vis, zeta potential) of Cu/MWCNT and Ag/MWCNT hybrid nanofluids prepared in EG using probe ultrasonication. Thermal conductivity was measured with the transient hot‐wire method at 20°C–70°C. The results are compared with existing data in Table 1 and discussed in the context of the previous Cu, Ag, and hybrid nanofluid literature.

## 2 Experiments and Results

### 2.1 Materials and Methods

Carboxyl‐functionalized multiwalled CNTs (MWCNT–COOH, 30–50 nm) were obtained from US Research Nanomaterials, Inc. and which were produced via chemical vapor deposition (CVD). The surface modification (COOH ≈ 2.3 at%) was verified by FTIR and the supplier's certification. Silver (Ag, 20 nm, >99%) and copper (Cu, 30 nm, >99%) nanoparticles were sourced from the same supplier and used as received. It should be noted that Ag and Cu nanoparticles were presynthesized materials and were not formed in situ during nanofluid preparation. Therefore, the probe‐ultrasonication step was used to disperse the premade nanoparticles and promote their attachment onto the carboxyl‐functionalized MWCNT surface, rather than to induce chemical nucleation, reduction, or growth.

**FIGURE 2:**
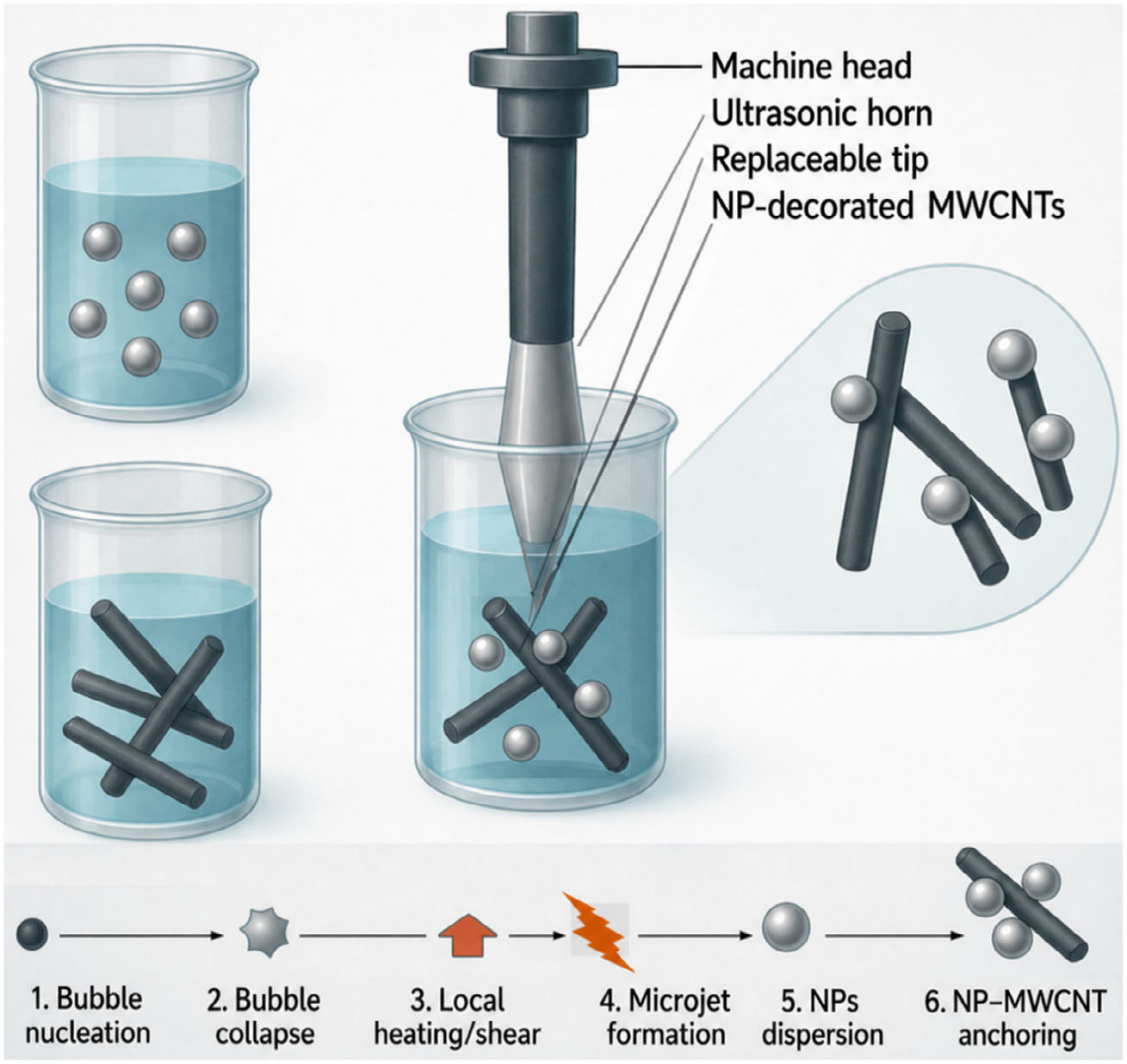
Illustration of synthesis of hybrid nanofluids using the ultrasonication mechanism.

EG was used as the base fluid. Hybrid nanofluids were prepared by dispersing 0.001 wt% MWCNTs with varying Ag and Cu concentrations. The suspensions were initially prestirred mechanically and ultrasonicated using a VCX‐750 processor (750 W, 20 kHz, 13 mm probe) operated in pulsed mode (2s on/2s off, 30% amplitude, 15 min). Temperature was maintained below 30°C using a thermal bath. These conditions ensured homogeneous energy distribution throughout the suspension and reproducible dispersion. The synthesis procedure is schematically illustrated in Figure 2.

**FIGURE 3:**
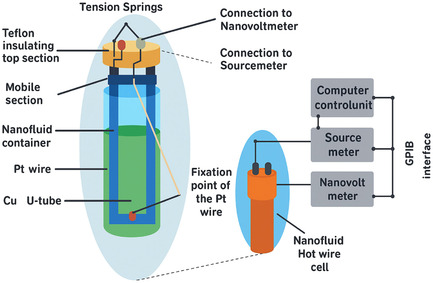
Schematic description of the standard hot‐wire setup for the thermal conductivity measurements.

Characterization was carried out using TEM (morphology and decoration), Raman spectroscopy (bonding structure), UV–Vis spectroscopy (plasmonic response and stability), and zeta‐potential measurements (surface charge and stability). Thermal conductivity was measured using the standard hot‐wire method, as schematically shown in Figure 3. The thermal conductivity measurements were carried out using a Thermtest THW1 analyzer based on the transient hot‐wire method with a platinum sensor. Prior to all nanofluid measurements, the system was calibrated using a reference deionized water sample provided by the manufacturer. The calibration procedure was performed three times, and the measured values showed excellent agreement with the reference data within the instrument's specified uncertainty (±2%). Temperature calibration was verified using the instrument software in conjunction with an external Watlow temperature controller. Following calibration, all nanofluid measurements were conducted under identical experimental conditions. Before each thermal conductivity measurement, the sample was held at the target temperature for approximately 30 min to ensure thermal equilibrium and minimize temperature gradients within the nanofluid. Accordingly, the term “decoration” in this work refers to the ultrasonication‐assisted attachment of presynthesized Ag or Cu nanoparticles onto MWCNT–COOH, not to in situ chemical growth or deposition from metal precursors.

### 2.2 Morphological Investigations

**FIGURE 4:**
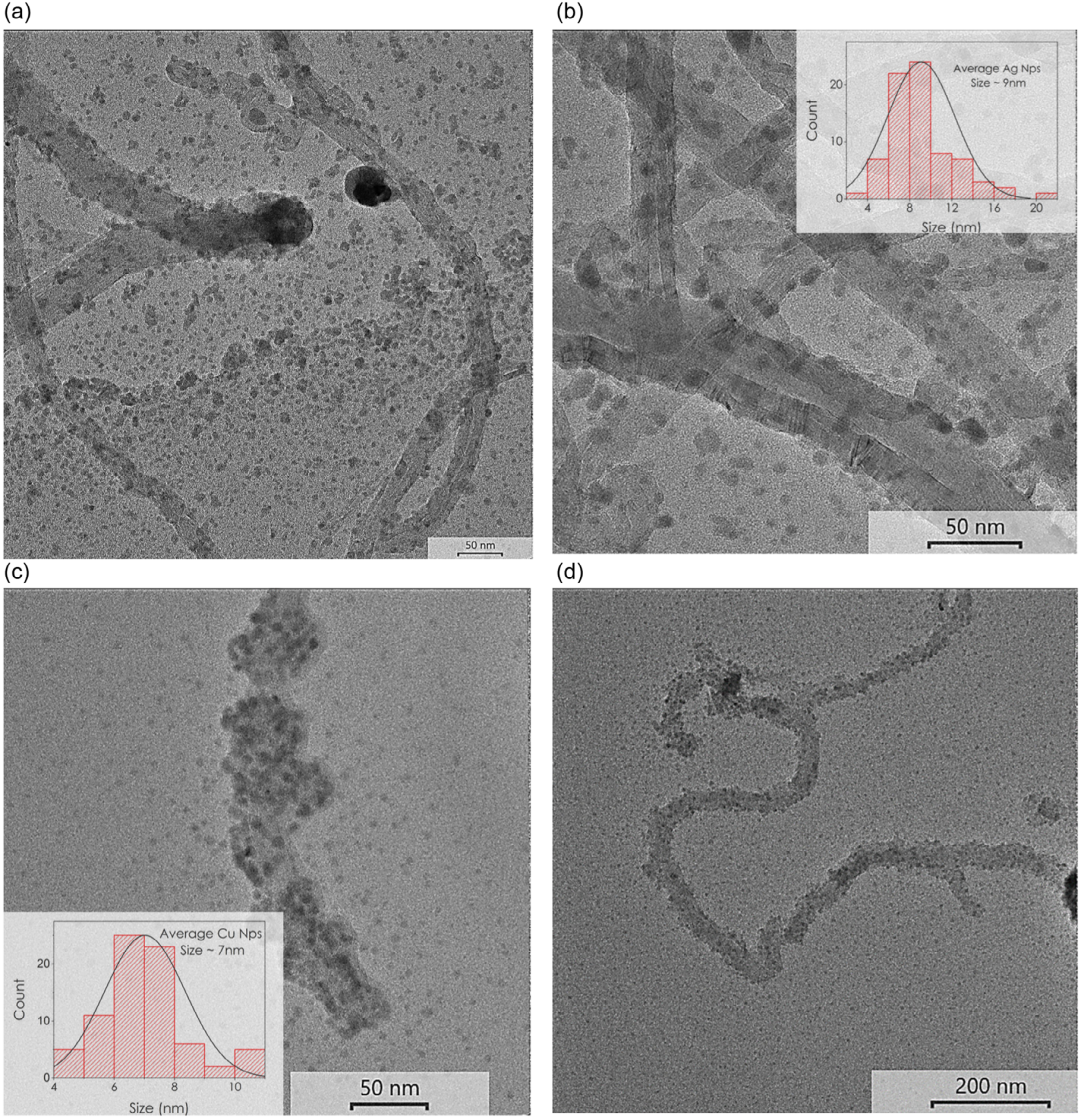
TEM micrographs of the synthesized nanocomposites: (a,b) Ag‐decorated MWCNTs showing uniformly dispersed Ag nanoparticles anchored along the nanotube walls and corresponding size distribution of Ag Nps with an average size of 9 nm and (c,d) Cu‐decorated MWCNTs exhibiting chain‐like Cu nanoparticle clusters distributed continuously along the MWCNT framework and corresponding size distribution of Cu Nps with an average size of 7 nm.

Morphological characterization was carried out using TEM to assess dispersion quality, structural integrity, and surface decoration of the synthesized metal‐MWCNT nanocomposites. TEM analysis provides morphological insight into the spatial distribution and morphology of Ag and Cu nanoparticles on the CNT framework, enabling the evaluation of nanoparticle size, anchoring uniformity, and potential agglomeration. These observations are essential for correlating the nanocomposites’ microstructural features with their anticipated physicochemical and thermal performance (see Figure 4).

From Figure 4, one could observe that both Ag‐ and Cu‐decorated MWCNTs exhibit well‐defined metal nanoparticle attachment along the nanotube surfaces. The Ag/MWCNT samples reveal uniformly dispersed spherical silver nanoparticles anchored across the nanotube walls with minimal aggregation, indicating effective dispersion and strong interfacial interactions. In comparison, the Cu/MWCNT nanocomposites display a chain‐like distribution of copper nanoparticles, forming continuous clusters along the nanotube length while preserving the structural flexibility of the carbon framework. In both cases, the nanotubes retain their characteristic tubular morphology, and the nanoparticles remain uniform in size and well‐distributed. These morphological features support metal nanoparticle distribution on the MWCNT framework and suggest that the synthesis strategy effectively promotes uniform metal concentration while minimizing particle agglomeration.

Comparatively, the Ag‐decorated MWCNTs exhibit more discrete, isolated nanoparticles, while the Cu‐decorated samples form more interconnected cluster structures. These differences arise from the distinct dispersion and attachment behaviors of the presynthesized metal nanoparticles on the MWCNT surface. Despite these distinctions, both materials show uniform decoration and good dispersion stability, confirming that the adopted synthesis route is effective for producing well‐anchored metal‐MWCNT nanocomposites suitable for advanced thermal and physicochemical applications.

The effective anchoring of metallic nanoparticles on the –COOH‐functionalized MWCNT framework can be attributed to a combination of electrostatic, van der Waals, and interfacial interactions occurring during dispersion and ultrasonication. The presence of surface carboxyl and hydroxyl groups provides polar interaction sites along the MWCNT walls, which can facilitate the attachment of presynthesized Ag and Cu nanoparticles. Under high‐energy ultrasonication, transient cavitation bubbles generate localized microjets and shear fields that break weak agglomerates, improve particle mobility, and promote the physical attachment of nanoparticles onto the nanotube surface.

For presynthesized metal nanoparticles, acoustic activation improves dispersion and particle mobility, increasing the probability of contact with oxygen‐functional sites on the carbon surface. Furthermore, the differential electronegativity between carbon and metallic species promotes partial charge transfer at the interface, improving adhesion and electronic coupling. For Ag/MWCNT composites, the noble nature of silver limits oxide formation, leading to stable metal–carbon interfaces with low interfacial resistance. In contrast, Cu nanoparticles tend to form thin surface oxide shells during dispersion, which can still interact favorably with polar –COOH groups through hydrogen bonding or van der Waals forces. These interactions support stable nanoparticle attachment without requiring additional surfactants or reducing agents.

Such interfacial association not only ensures long‐term colloidal stability of the hybrid nanofluids but also enhances phonon bridging across the metal–carbon interfaces, which is a key factor governing thermal conductivity enhancement. The combined effects of strong interfacial coupling, nanoscale contact area, and preserved MWCNT integrity contribute to efficient heat transport and the overall enhanced thermophysical performance of the synthesized Cu/MWCNT and Ag/MWCNT hybrid nanofluids.

### 2.3 Raman Spectroscopy

**FIGURE 5:**
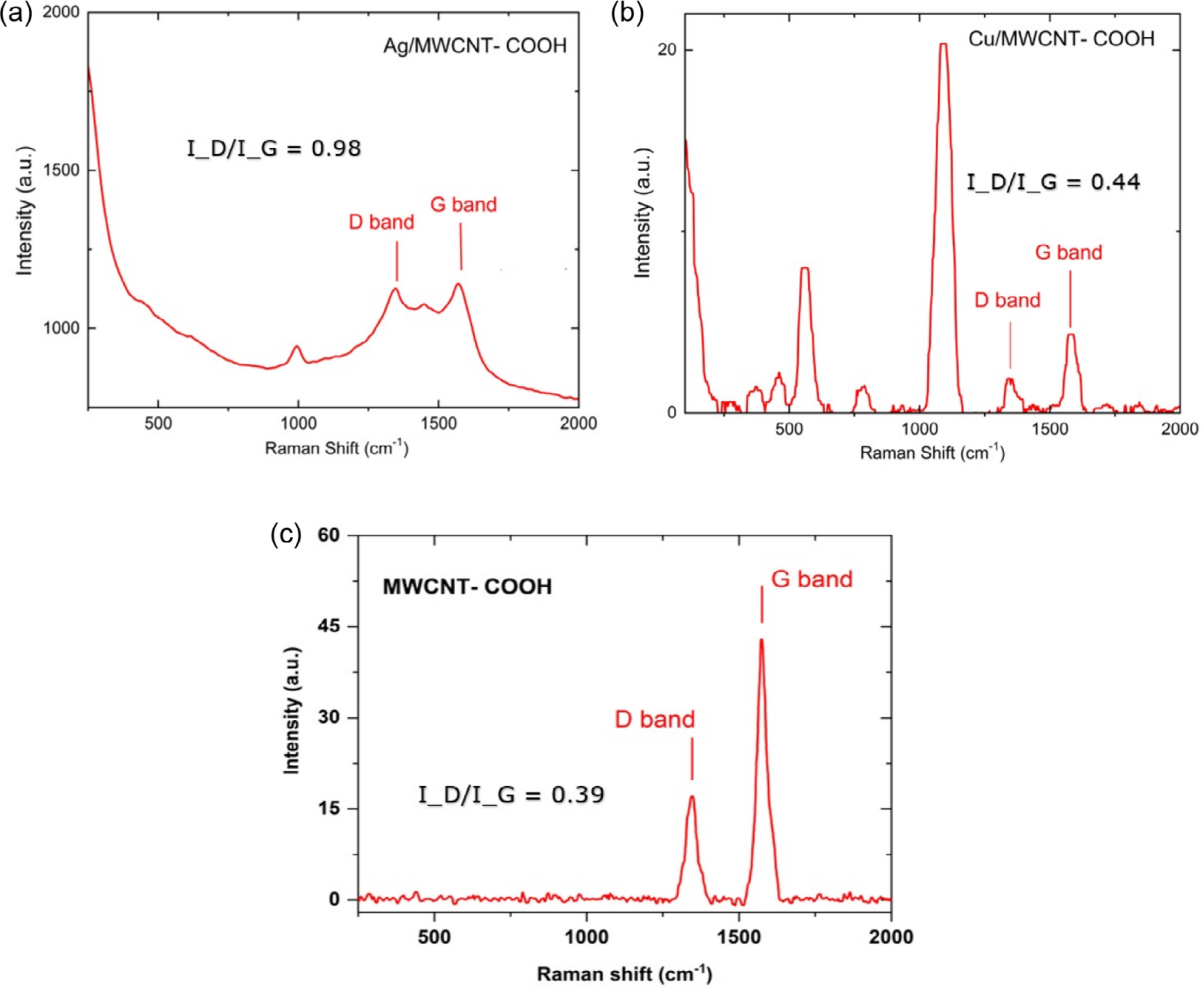
Raman spectrum of (a) Ag/MWCNTs, (b) Cu/MWCNT nanocomposites, and (c) MWCNT.

The structural and vibrational characteristics of the synthesized nanocomposites were analyzed by Raman spectroscopy, as presented in Figure 5. For comparison, the Raman spectrum of MWCNT–COOH powder was also included as a reference to evaluate changes induced by Ag and Cu nanoparticle incorporation. Both Ag/MWCNT–COOH and Cu/MWCNT–COOH samples exhibit two characteristic peaks corresponding to the D‐band (≈1330–1350 cm^−1^) and the G‐band (≈1570–1590 cm^−1^), which are typical signatures of MWCNTs. The D‐band arises from the breathing mode of sp^2^‐hybridized carbon atoms at lattice defects or disordered regions, while the G‐band originates from the in‐plane stretching vibration of the C—C bonds within the graphitic domains.

A change in relative intensity ratio (*I*_D_/*I*_G_) was observed in both composites compared to MWCNT–COOH, suggesting structural perturbation of the MWCNT framework after incorporation of the metal nanoparticles. The increase in *I*_D_/*I*_G_ from roughly 0.39 for MWCNT–COOH to 0.44 for Cu/MWCNT and to 0.98 for Ag/MWCNT, suggesting an increased defect‐related Raman contribution after interaction with the metal nanoparticles. This suggests possible defect‐mediated interaction between the metallic nanoparticles and oxygenated functional groups or defect sites on the nanotube walls.

In the Ag/MWCNT spectrum, the reduced D‐band width may indicate partial restoration of graphitic ordering or a change in defect distribution, likely caused by charge transfer between Ag and the conjugated π‐electron system of MWCNTs. Conversely, the Cu/MWCNT spectrum shows a marginal broadening of both D and G bands, which may be attributed to with local lattice strain and possible interaction between Cu‐containing surface species and oxygenated functional groups during sonication. The absence of clear additional Raman peaks related to CuO or Ag_2_O phases suggests that no dominant oxide phase was detected by Raman spectroscopy under the present measurement conditions.

These Raman features collectively suggest that Ag and Cu nanoparticles interact with the MWCNT matrix through surface defect sites and oxygenated functionalities, supporting the enhanced defect‐mediated interfacial interaction between the metal nanoparticles and the functionalized MWCNT framework without evidence of significant structural degradation. Such defect‐mediated interactions may contribute to improved phonon coupling and interfacial thermal transport in the resulting hybrid nanofluids.

### 2.4 UV–Vis Absorbance and Stability

**FIGURE 6:**
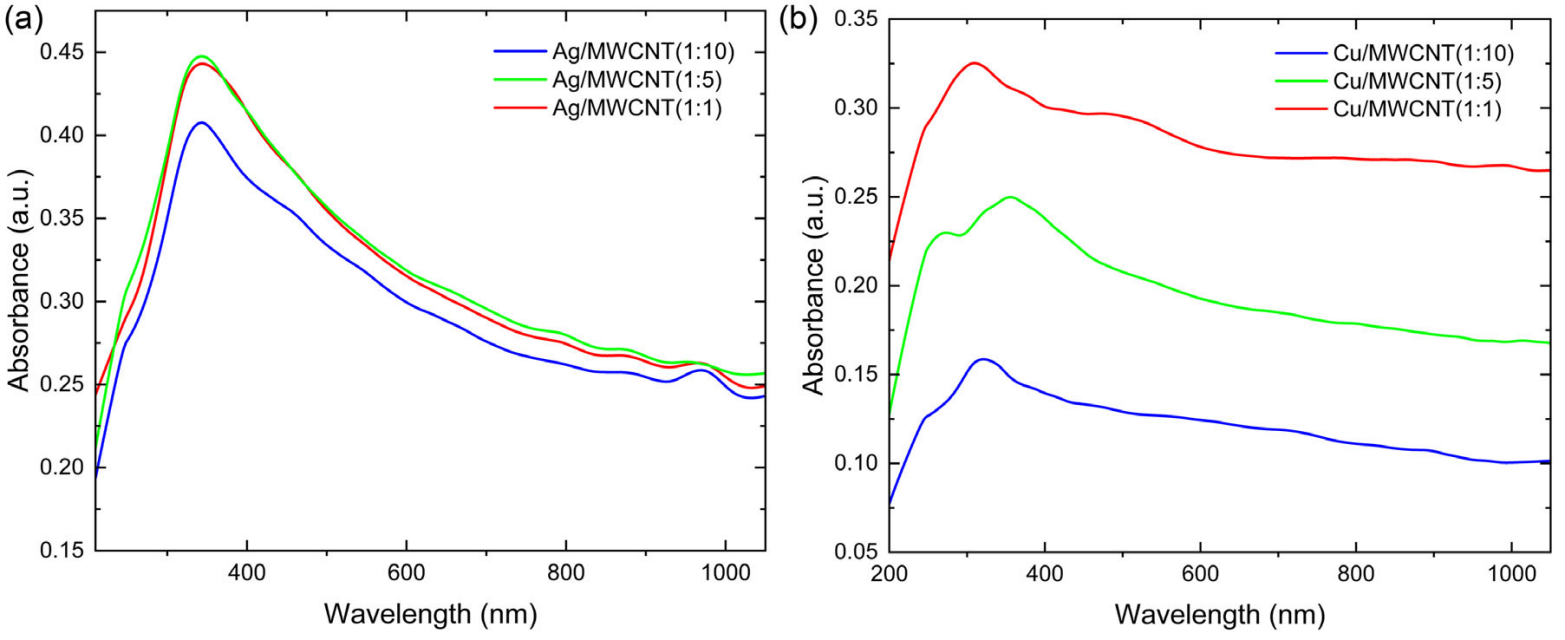
UV–Vis absorption spectra of (a) Ag/MWCNT and (b) Cu/MWCNT nanocomposites prepared with fixed MWCNT concentration (0.001 wt%) and varying metal concentrations of 0.001, 0.005, and 0.01 wt% in ethylene glycol. Increasing metal content results in systematic absorbance enhancement with minimal spectral shifting, confirming stable dispersion and effective metal decoration of the MWCNT framework.

The optical absorption spectra of the Ag/MWCNT and Cu/MWCNT hybrid nanocomposites indicate the successful formation of metal‐decorated nanotube structures and their stable dispersion in EG. As shown in Figure 6, the Ag/MWCNT samples exhibit a clear plasmonic feature in the 380–420 nm region, with the most pronounced peak observed at the highest Ag concentration (0.01 wt%). This band corresponds to the localized surface plasmon resonance (LSPR) of Ag nanoparticles, supporting the presence of nanoscale Ag particles and their stable dispersion within the MWCNT–EG suspension. In contrast, the Cu/MWCNT spectra display a broader and blue‐shifted absorption band centered around 300–360 nm. The reduced intensity and broader line shape are characteristic of Cu nanoparticles due to stronger electron–phonon damping and the partial formation of Cu_2_O/CuO surface layers during ultrasonication‐assisted synthesis.

Increasing the metal concentration (0.001 → 0.005 → 0.01 wt%) leads to a systematic enhancement in absorbance for both Ag and Cu systems. The spectral shapes remain consistent across concentrations, indicating that no significant agglomeration or interparticle coupling occurs at higher concentrations. The nearly featureless background extending into the visible and near‐IR regions confirms that the MWCNT framework remains well dispersed and introduces minimal optical interference in this range. Overall, the UV–Vis spectra provide strong supporting evidence of dispersion of the presynthesized metal nanoparticles and stable attachment to MWCNTs within EG.

A direct comparison between the Ag/MWCNT and Cu/MWCNT spectra reveals distinct plasmonic behaviors arising from the intrinsic optical properties of the two metals. Ag/MWCNTs exhibit a sharper and more clearly defined LSPR peak, reflecting the lower damping and stronger plasmonic response of silver nanoparticles. The peak intensity increases approximately linearly with increasing Ag concentration, indicating concentration‐dependent absorbance and effective nanoparticle dispersion. In contrast, the Cu/MWCNT spectra show broader, less intense plasmonic bands that are blueshifted, consistent with the inherently weaker and more easily damped LSPR of copper. The more pronounced broadening in Cu/MWCNTs suggests partial surface oxidation, which is expected for Cu nanoparticles in polar solvents. Despite these differences, both systems show monotonic intensity increases with metal concentration and stable spectral profiles with no evidence of significant agglomeration. These distinctions indicate that while both metals are successfully incorporated onto the MWCNTs, Ag produces a stronger and more defined plasmonic signature, whereas Cu exhibits a more damped, oxidation‐influenced optical response.

**FIGURE 7:**
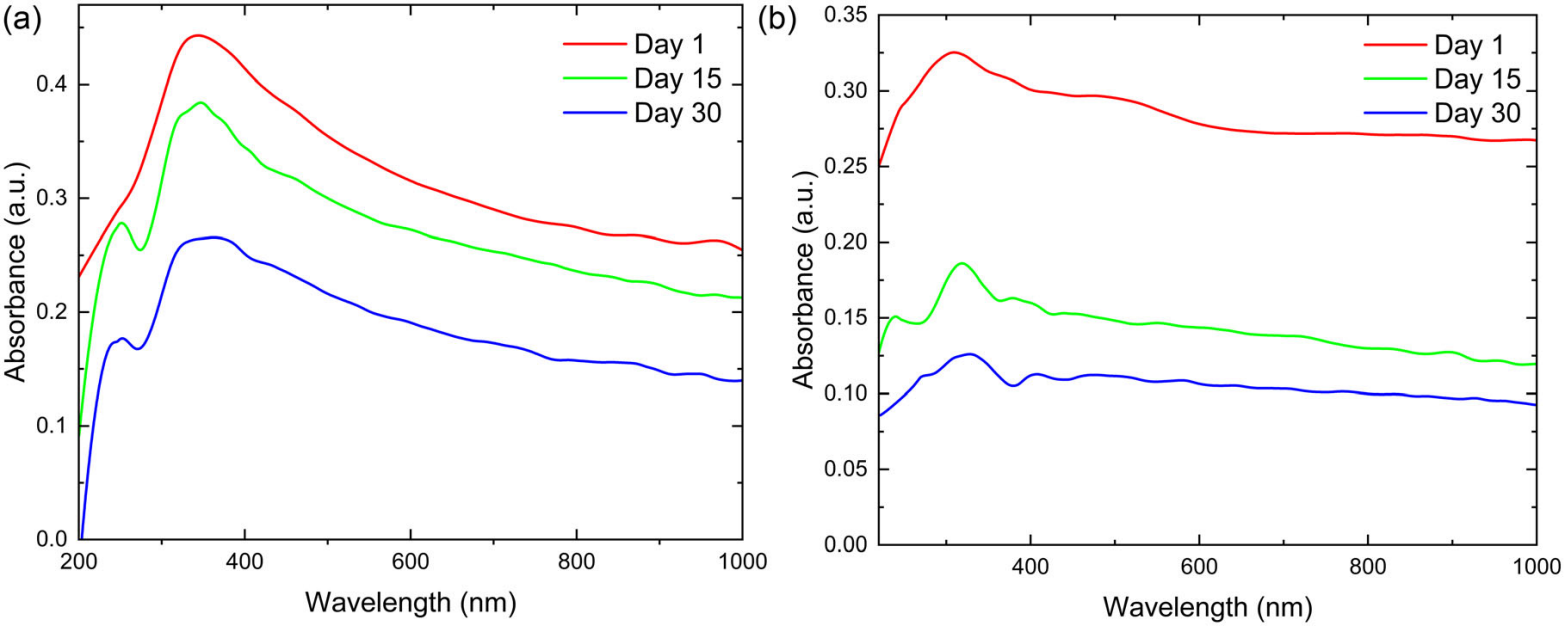
UV spectra of (a) Ag/MWCNT and (b) Cu/MWCNT nanofluids measured after 1, 15, and 30 days of storage at room temperature. Both nanofluids have the lowest metal concentration of 0.001 and 0.001 wt% of MWCNTs.

On the other hand, for the lowest metal concentration (0.001 wt%), the long‐term colloidal stability of the Ag/MWCNT–EG and Cu/MWCNT–EG nanofluids was assessed using time‐resolved UV–Vis spectroscopy and zeta‐potential measurements over a 30‐day storage period. The 30‐day stability analysis was performed at the lowest metal loading (0.001 wt%) because the main objective of this work was to evaluate the feasibility of stable hybrid nanofluids at ultralow concentrations. This formulation represents the most dilute sample investigated and is expected to impose the lowest viscosity and pumping‐power penalty, which is important for thermal management systems. As shown in Figure 7, both nanofluids exhibit a gradual decline in overall absorbance intensity from Day 1 to Day 30. This decrease is consistent with mild sedimentation or concentration loss over time, although quantitative sedimentation analysis was not performed. This is supported by the characteristic spectral shapes and plasmonic peak positions, which remain essentially unchanged. The absence of peak broadening, shifting, or new spectral features indicates that neither system shows evidence of significant nanoparticle growth, agglomeration, or chemical transformation during storage.

For the Ag/MWCNT–EG nanofluid, the distinct silver plasmonic band in the 380–420 nm region preserves its shape even after 1 month. Although peak intensity decreases, the retention of a sharp profile indicates that Ag nanoparticles remain well dispersed on the MWCNT framework with minimal interparticle coupling. In contrast, the Cu/MWCNT–EG nanofluid displays a broader, blueshifted plasmonic, damped absorption band around 300–360 nm, characteristic of copper nanoparticles with partial surface oxidation. While the absorbance also decreases progressively, the overall spectral shape remains unchanged, demonstrating that the Cu nanoparticles retain their morphology without significant additional oxidation or aggregation over time.

A direct comparison between the two nanofluids shows that Ag/MWCNT–EG maintains higher absorbance intensities and exhibits slower spectral decay compared to Cu/MWCNT–EG. This is consistent with the inherently higher plasmonic stability of Ag and its lower susceptibility to oxidative damping. Cu/MWCNT–EG shows a more pronounced reduction in absorbance but still preserves its spectral signature, suggesting that the decrease is more likely associated with mild sedimentation or concentration loss rather than severe aggregation or chemical degradation. Both systems therefore demonstrate good optical stability over the 30 day period.

**FIGURE 8:**
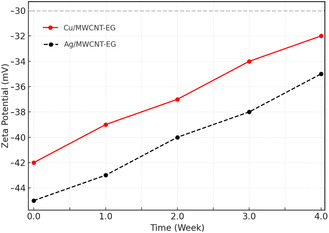
Time‐dependent zeta‐potential values of Ag/MWCNT–EG and Cu/MWCNT–EG nanofluids monitored over 4 weeks. Both nanofluids retain zeta‐potential magnitudes |*ζ*| ≥ 30 mV. Both nanofluids have the lowest metal concentration of 0.001 and 0.001 wt% of MWCNTs.

The optical stability correlates with high‐magnitude zeta potential (|*ζ*| ≥ 30 mV), as shown in Figure 8. Both Ag/MWCNT–EG and Cu/MWCNT–EG retain zeta‐potential magnitudes above the stability threshold across 4 weeks. This confirms a strong surface charge that promotes sustained colloidal dispersion. Such stability, achieved without surfactants, can be attributed to the combined effects of carboxyl functionalization of MWCNTs, localized shear forces during probe ultrasonication, and the resulting metal–carbon interfacial bonding. Overall, UV–Vis spectroscopy thus provides strong supporting evidence of homogeneous dispersion of the presynthesized metal nanoparticles and the month‐scale colloidal stability of the synthesized nanofluids, making them suitable for practical thermal management applications. It is worth noting that although quantitative sedimentation measurements or time‐resolved vial photographs were not investigated, the preserved spectral shape and zeta‐potential magnitude after storage suggest that the observed absorbance decrease is not associated with severe aggregation or chemical transformation.

### 2.5 Thermal Conductivity

**FIGURE 9:**
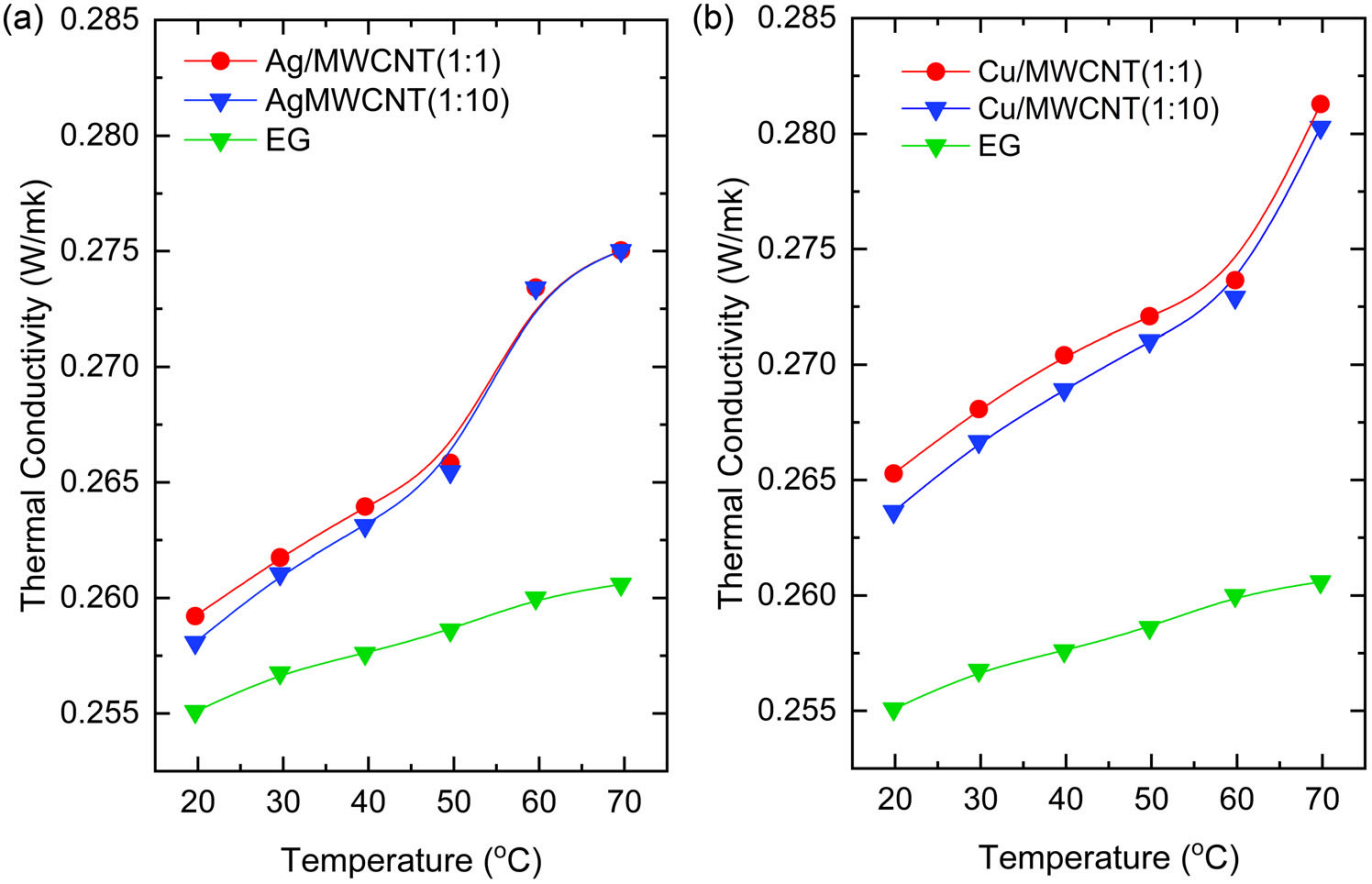
Temperature‐dependent thermal conductivity of (a) Ag/MWCNT–EG and (b) Cu/MWCNT–EG hybrid nanofluids measured at two metal concentration (0.001 and 0.01 wt%) in comparison with pure ethylene glycol.

The thermal transport behavior of Ag/MWCNT–EG and Cu/MWCNT–EG hybrid nanofluids was evaluated over the temperature range of 20°C–70°C using the transient hot‐wire technique (see Figure 9). The reported thermal conductivity trends were obtained after calibration verification and temperature equilibration at each measurement point; the measurement uncertainty of the THW1 system was within ±2%. This uncertainty is primarily associated with probe calibration, temperature control, and signal acquisition in the transient hot‐wire system, based on the instrument specifications (Thermtest THW1). Both hybrid systems exhibit a clear and monotonic increase in thermal conductivity with rising temperature and metal nanoparticle concentration. The smooth and monotonic variation of thermal conductivity with temperature and nanoparticle concentration, together with the prior calibration of the measurement system, supports the consistency and reliability of the obtained results.

**FIGURE 10:**
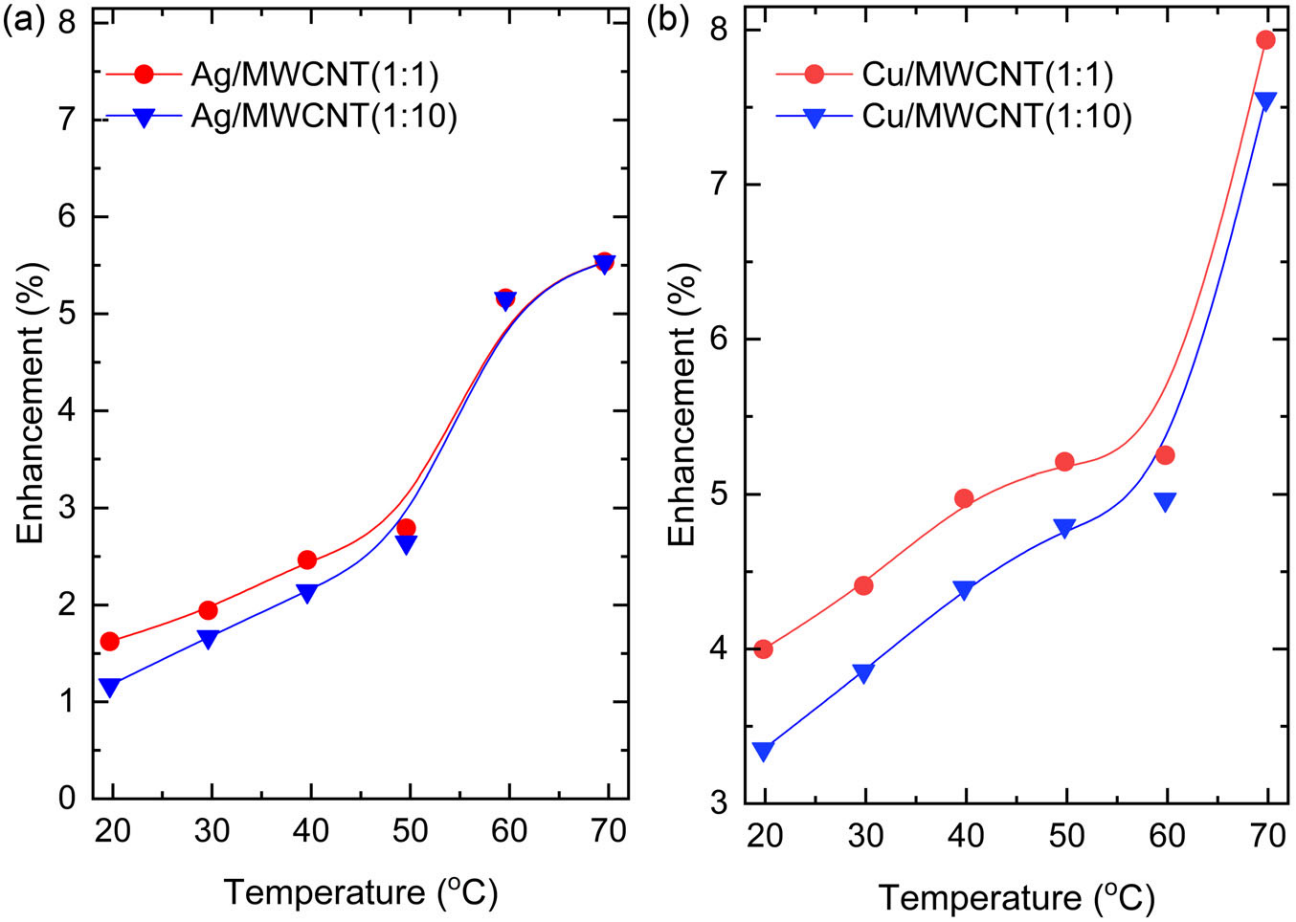
Percentage enhancement in thermal conductivity of (a) Ag/MWCNT–EG and (b) Cu/MWCNT–EG nanofluids as a function of temperature at two metal concentrations (0.001 and 0.01 wt%). Enhancement increases sharply at elevated temperatures due to thermally activated Brownian effects.

Even at the ultralow concentration of 0.001 wt% metal, the nanofluids demonstrate measurable improvements of approximately 2%–3% relative to pure EG at low temperatures. Increasing the metal content to 0.01 wt% leads to substantial enhancements, reaching ~5.5% for Ag/MWCNT–EG and ~8% for Cu/MWCNT–EG at 70°C (see Figure 10).

The temperature‐dependent enhancement can be attributed to intensified Brownian motion, possible microconvective effects, and decreased base fluid viscosity at elevated temperatures, which collectively improve the rate of nanoparticle‐fluid energy exchange. The sharp rise in enhancement between 50°C–60°C for Ag/MWCNT–EG and 60°C–70°C for Cu/MWCNT–EG reflects thermally activated mobility of the suspended hybrid structures, increasing the number of effective collisions and conductive pathways within the fluid.

**FIGURE 11:**
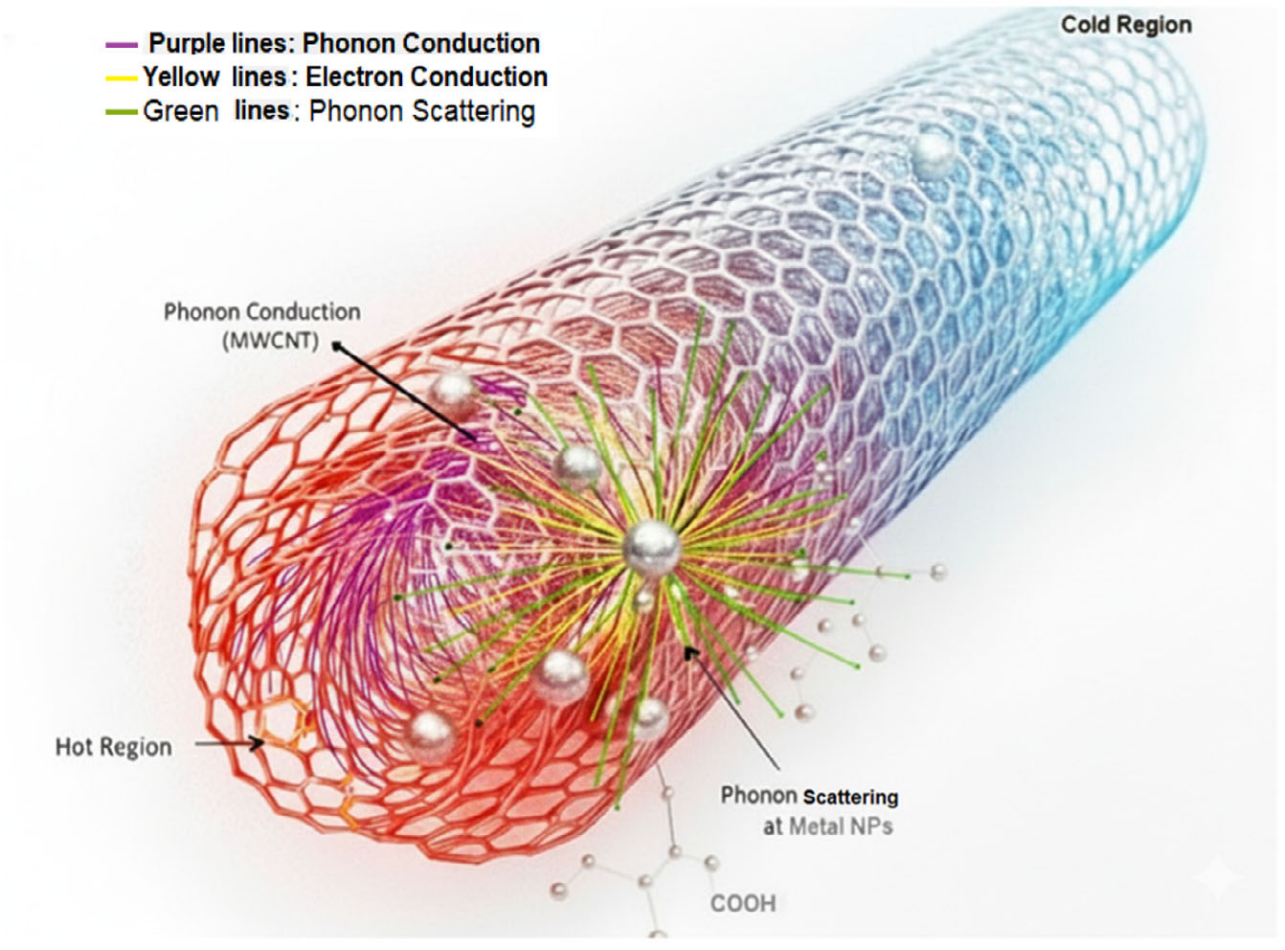
Schematic representation of the synergistic heat transfer mechanisms in the metal/MWCNT hybrid nanofluid. The overall heat flux is enhanced by phonon conduction along the MWCNT lattice (purple paths); electronic conduction through the decorating metal nanoparticles (orange paths) and phonon scattering (green paths).

Beyond Brownian dynamics, the hybrid nanostructure may play a significant role in heat transport performance. Probe ultrasonication promotes dispersion and possible interfacial association of Ag or Cu nanoparticles onto functionalized MWCNT surfaces, supporting possible phonon–electron heat‐transfer pathways. The MWCNTs serve as one‐dimensional thermal bridges, while the attached metal nanoparticles provide additional interfacial sites that decrease Kapitza resistance and enable parallel channels for phonon and electron‐assisted heat conduction [3, 4, 5, 18]. The enhanced thermal conductivity (*k*) may arise from several synergistic mechanisms (see Figure 11). Heat is transferred from the hot region to the cold region via two parallel solid‐state conduction pathways: high‐speed phonon conduction along the MWCNT lattice (purple paths) and electronic conduction through the decorating metal nanoparticles (Yellow paths). However, the interface between the metal nanoparticle and the MWCNT introduces thermal boundary resistance (*R*_TBR_) or Kapitza resistance, which impedes heat flow due to phonon mismatch. This impedance, along with internal structural defects, causes significant phonon scattering (green paths). Further heat transfer within the bulk fluid is facilitated by the motion of the suspended particles, including Brownian motion of the hybrid structures, the possible formation of interfacial liquid layers (nanolayer) of EG molecules around the particle surfaces, and the possible development of percolation‐like thermal pathways across the fluid. The hybrid structure may promote effective thermal contacts without increasing viscosity or triggering sedimentation.

Although Ag possesses one of the highest intrinsic thermal conductivities among common metals, the Cu/MWCNT–EG nanofluid exhibits slightly superior enhancement. This outcome may be associated with stronger interfacial interaction between Cu nanoparticles and the oxygenated –COOH groups on MWCNTs, which may reduce interfacial resistance and improve heat transfer across the hybrid structure. The Ag/MWCNT interface, while stable, exhibits comparatively weaker interfacial interaction, leading to slightly higher interfacial resistance and lower overall conductivity enhancement. It should be noted that these mechanisms are proposed to explain the observed thermal conductivity trends, and their individual contributions were not independently quantified in the present study.

As summarized in Table 1, the observed enhancement levels are notable when compared with previous studies performed at higher nanoparticle concentrations. Earlier works have typically required approximately 0.05–0.3 wt% or higher loadings to achieve comparable thermal conductivity improvements, including Cu/EG systems (Garg et al. [43]), Ag‐decorated CNTs (Munkhbayar et al. [50]; Farbod and Ahangarpour [49]), Cu/Ag hybrids (Babar et al. [48]), and CuO/graphene composites (Kumar et al. [53]). In contrast, the present nanofluids deliver up to 8% enhancement at only 0.01 wt% metal concentration, which is lower than the typical wt% loadings reported in comparable studies, highlighting the high thermal efficiency of the surfactant‐free metal–MWCNT hybrid architecture.

These properties make Cu/MWCNT–EG and Ag/MWCNT–EG nanofluids promising candidates for low‐concentration, energy‐efficient heat transfer fluids applicable in compact heat exchangers, solar thermal absorption loops, and electronic cooling systems.

Building upon the confirmed month‐scale stability, subsequent research will focus on assessing the long‐term durability of these nanofluids. Future studies will include detailed analysis of thermal conductivity retention over extended periods and the long‐term sustainability and industrial scalability of the fabrication route.

## 3 Conclusion

Ag/MWCNT and Cu/MWCNT hybrid nanofluids were successfully prepared in EG using a surfactant‐free probe ultrasonication route, enabling the attachment of presynthesized metallic nanoparticles onto carboxyl‐functionalized MWCNTs. The combined characterization results supported the formation of well‐dispersed metal/MWCNT hybrid suspensions, while the main outcome of the study was the achievement of measurable thermal conductivity enhancement at ultralow particle loadings. For the lowest metal concentration (0.001 wt%), time‐resolved UV–Vis and zeta‐potential analyses demonstrated acceptable month‐scale colloidal stability, with preserved spectral features and |*ζ*| ≥ 30 mV after 4 weeks, despite a gradual absorbance decrease attributed to mild sedimentation or concentration loss.

Thermal conductivity increased systematically with both nanoparticle content and temperature, achieving thermal conductivity enhancements of ≈5.5% and ≈8% for Ag/MWCNT–EG and Cu/MWCNT–EG, respectively, at 0.01 wt% solids and 70°C. The stronger performance of Cu/MWCNT–EG may be associated with improved interfacial interaction between Cu nanoparticles and oxygenated defect sites on the MWCNTs, which could contribute to more effective heat transfer across the hybrid structure. The hybrid metal–MWCNT structure is expected to support conductive pathways that contribute to the observed thermal enhancement.

Relative to existing nanofluid studies, which typically require approximately 0.05–0.3 wt% solids or higher to achieve similar gains, the present results demonstrate that tailored interfacial engineering at ultralow concentrations can deliver competitive thermal transport performance without significant viscosity rise or sedimentation. These findings highlight the potential of Ag/MWCNT–EG and Cu/MWCNT–EG nanofluids as low‐concentration, energy‐efficient heat transfer fluids for high‐heat‐flux heat exchangers and electronic cooling applications, where reduced nanoparticle concentration, acceptable dispersion stability, and lower expected viscosity/pumping penalties are important practical advantages. Future work may explore tuning Cu‐Ag ratios, rheological behavior, and coupling with semiconductor nanostructures to further optimize optothermal functionality. Additionally, future work should include dedicated heating–cooling cycle tests to evaluate the long‐term thermophysical stability of the proposed nanofluids.

## Conflicts of Interest

The authors declare no conflicts of interest.

## Data Availability

All data generated or analyzed during this study are included in the manuscript.
